# Application of radial artery signal characteristics in long-term head-down bed rest experimental cardiovascular status assessment

**DOI:** 10.3389/fphys.2026.1961052

**Published:** 2026-10-21

**Authors:** Kaiting Ha, Xiaojuan Hu, Dong Liu, Guohao Zhang, Shuyi Zhang, Shiju Zhao, Liping Tu, Tao Jiang, Xuxiang Ma, Quanchun Fan, Junlian Liu, Chengpu Zhang, Hongzhi Shi, Jiatuo Xu, Ji Cui

**Affiliations:** 1 School of Traditional Chinese Medicine, Shanghai University of Traditional Chinese Medicine, Shanghai, China; 2 School of Artificial Intelligence of Traditional Chinese Medicine, Shanghai University of Traditional Chinese Medicine, Shanghai, China; 3 National Key Laboratory of Space Medicine, China Astronaut Research and Training Center, Beijing, China; 4 School of Integrative Medicine, Shanghai University of Traditional Chinese Medicine, Shanghai, China

**Keywords:** -6° head-down bed rest, cardiovascular ultrasound indicators, dynamic monitoring, simulated weightlessness, TCM pulse diagnosis

## Abstract

**Objective:**

This study aims to observe pulse graph parameters and their dynamic changes in subjects of the head-down bed rest (HDBR) under different exercise interventions and to evaluate their application value in simulating cardiovascular function changes in weightlessness.

**Methods:**

A total of 36 male volunteers (32.0 ± 2.6 years) participated in a 138-day experiment (15 days at baseline, 90 days at -6° HDBR, and 33 days at recovery). Radial pulse graphs were collected at 11 time points: the baseline, D7, D15, D30, D45, D60, D75, D90 during bedrest, and R + 7, R + 15, and R + 30 during the recovery. Cardiac structural and functional parameters, along with arterial elasticity and blood flow parameters, were recorded simultaneously. The feasibility of TCM pulse diagnosis for assessing cardiovascular status in HDBR subjects was explored through correlation analyses between pulse parameters and ultrasound indicators.

**Results:**

(1) During bedrest, pulse parameters h_1_, h_3_, h_4_, t_5_, t, As, Ad, and h_4_/h_1_ showed an upward trend and decreased during the recovery stage. PWVρ, crPWV, LICAvmax, and LFAvmax increased initially and then decreased, whereas IMT showed a sustained increase. (2) The aerobic exercise plus resistance training group showed faster adaption to HDBR and better cardiovascular functional recovery after long-term HDBR. (3) Canonical correlation and bivariate correlation analyses showed varying degrees of correlation between the two types of indicators, with some correlation coefficients >0.7 (*P* < 0.05). (4) Repeated measurements showed statistically significant differences in pulse graph parameters, with cfPWV, crPWV, and PWVρ showing an increasing trend followed by a decrease.

**Conclusion:**

Cardiovascular changes induced by HDBR can be reflected in pulse graph parameters; radial pulse wave parameters may serve as valuable references for the dynamic monitoring and assessment of cardiovascular function under simulated weightlessness.

## 1 Introduction

The completion of the Chinese space station has ushered in a new era of long-term space habitation, and the extended duration of astronaut missions in orbit has made the health impacts of weightlessness increasingly prominent. Under microgravity conditions, body fluids redistribute and shift upward, leading to facial swelling, muscle atrophy, bone loss, and a series of other physiological changes (Kamiya et al., 2000; Aubert et al., 2005; Pavy-Le Traon et al., 2007; Jirak et al., 2022). The impact on the cardiovascular system is particularly significant, manifested as elevated central venous pressure and increased cardiac preload, which, in turn, triggers compensatory changes in stroke volume and cardiac output; meanwhile, the transmural pressure and hemodynamic forces on peripheral vessel walls such as the carotid and femoral arteries also undergo corresponding changes, manifested as accelerated blood flow velocity and enhanced vascular tension in the upper body while the lower body exhibits the opposite pattern with reduced perfusion and decreased vascular resistance as vasomotor function and peripheral resistance undergo adaptive readjustment (Amirova et al., 2020; Xu et al., 2020; Boutouyrie et al., 2022; Rabineau et al., 2022; Scott et al., 2022). Therefore, assessing and counteracting the cardiovascular effects of weightlessness is essential for maintaining astronaut health during long-duration missions.

Currently, head-down bed rest (HDBR) is an internationally recognized ground-based model for studying the physiological effects of spaceflight, but effective countermeasures against cardiovascular deconditioning remain to be developed (Convertino and Sandler, 1995; Robin et al., 2022). In recent years, with the modernization of traditional Chinese medicine, pulse diagnosis technology has demonstrated potential application value in aerospace medicine, owing to its advantages in non-invasive dynamic monitoring (Tong et al., 1989; Yang et al., 2003; Liu et al., 2014; Liu et al., 2014; Li et al., 2015; Li et al., 2016; Shi et al., 2020). From the perspective of traditional Chinese medicine, the redistribution of body fluids and hemodynamic changes induced by weightlessness can be understood as a deviation of “qi” and “blood” from their normal flow state—changes in fluid volume affect the capacity of “qi” to propel blood circulation, while alterations in the mechanical environment of the vessel wall manifest as abnormal elasticity and blood flow velocity. Modern pulse diagnosis studies convert radial pressure waveforms into a series of quantitative parameters and assign them specific hemodynamic meanings (Fei, 2003). Therefore, this study utilizes “Cunkou” radial artery pulse parameters to observe dynamic pulse wave changes in subjects undergoing different exercise interventions during HDBR. We further explore the correlation between these pulse parameters and cardiovascular ultrasound indicators and evaluate the feasibility of using pulse wave parameters to assess cardiovascular status under simulated weightlessness.

## 2 Materials and methods

### 2.1 Research subjects

A total of 36 male volunteers, of Chinese nationality, were included in the study through social recruitment. Their average age was 32.0 ± 2.6 years; they had a junior high school education level or above and were in good health, with no tobacco or alcohol addiction, certain exercise habits, no drug dependence, no history of mental illness, psychiatric, genetic, and infectious diseases, no organic diseases (with special attention to bone and cardiovascular system diseases) or regional diseases, and no history of severe allergies. All of the contents of the experiment were reviewed and approved by the Medical Ethics Committee of the China Astronaut Research and Training Center (ethics no. ACC201904), and the volunteers signed a written informed consent form. The research process is shown in **Figure 1**.

**Figure 1:**
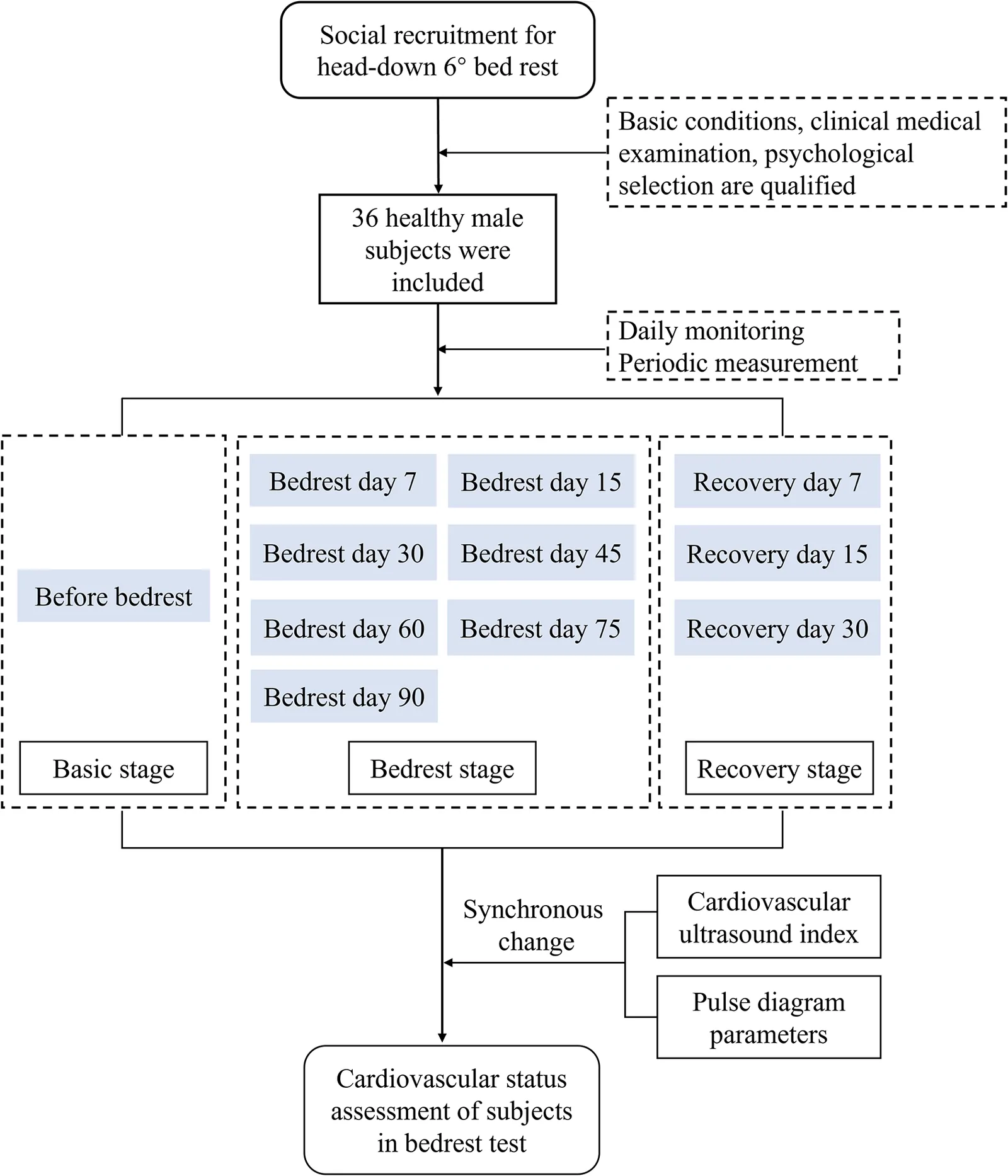
Flowchart of the HDBR pulse diagnostic evaluation study.

### 2.2 Experimental design

In this experiment, 36 subjects were randomly divided into five groups according to age, height, weight, peak oxygen consumption, maximum isometric strength during squats, and femoral neck bone mineral density (Wang et al., 2023): (1) control group (CON, *n* = 8, HDBR without countermeasure), (2) RUN group (RUN, *n* = 7, HDBR with aerobic exercises), (3) RES group (RES, *n* = 7, HDBR with resistance exercises), (4) C1 group, (C1, *n* = 7, HDBR with concurrent aerobic and lower-load resistance exercises), and (5) C2 group (C2, *n* = 7, HDBR with concurrent aerobic and high-load resistance exercises).

The experimental period lasts for a total of 138 days. The 15 days before bed rest are the pre-adaptation and basic data collection period. They had strict -6°HDBR for 90 days. The 33 days after rising up are the concentrated recovery period. Volunteers in bed maintained a -6° head position throughout, completing activities such as defecation, urination, dining, washing up, sleeping, testing, and transfer. They were allowed to change positions along the longitudinal axis of the body. During bedrest, blood pressure, heart rate, body temperature, water intake, urination, and general health are monitored daily.

### 2.3 Data measurement and analysis

In this study, the pulse diagnosis data collection unit of the DS01-T digital TCM four-diagnosis instrument was used to collect pulse diagnosis data, and the cardiovascular ultrasound indicators were measured using an ultrasound diagnostic instrument (Mindray Resona7). The volunteers were asked to rest for a moment before collection, maintain a -6° head down position, stretch their left forearm relaxed, palm up, place their wrist on the pulse pillow, flex their elbows to approximately 120°, and keep their bodies upright as well as breathe steadily, avoiding talking and body shaking. The pulse probe was placed on the subject’s left hand (high bone position behind the palm), the pulse graph amplitude was maximized, and the waveform was stabilized for 30 s to obtain the best pulse graph. Pulse diagnosis measurements were conducted at baseline before bedrest and then at uniform time points on days 7, 15, 30, 45, 60, 75, and 90 during the bedrest stage, as well as on days 7, 15, and 30 during the recovery stage.

The parameters of the pulse diagram mainly refer to the “Modern Chinese Medicine Pulse Diagnosis” edited by Fei Zhaofu (Scott et al., 2022), and the pulse graph analysis uses the time domain analysis method to analyze the relationship between the height of the pulse amplitude and the pulse phase. The parameters of the pulse graph are the wave of the pulse graph, the height of the gorge (h), the corresponding time value (t), etc., and the radial artery waveform data that have been preprocessed and segmented are then extracted from the pulse graph quantification parameters by using the fragrant energy envelope and Hilbert transform. The clinical significance of the corresponding parameters is shown in **Figure 2** and **Table 1**. The clinical significance of the cardiovascular ultrasound index parameters is shown in **Table 2**. In this study, the identification of outliers was carried out using the 2σ principle, which is defined as a set of measured values that deviate more than 2 standard deviations from the mean. The outlier value is handled by deleting the abnormal data and filling it by the last observed value carryover (LOCF), and the mean value of the corresponding state of the group is filled by the mean value of the corresponding state of the base state, 7 days of bedrest, and 7 days of recovery.

**Figure 2:**
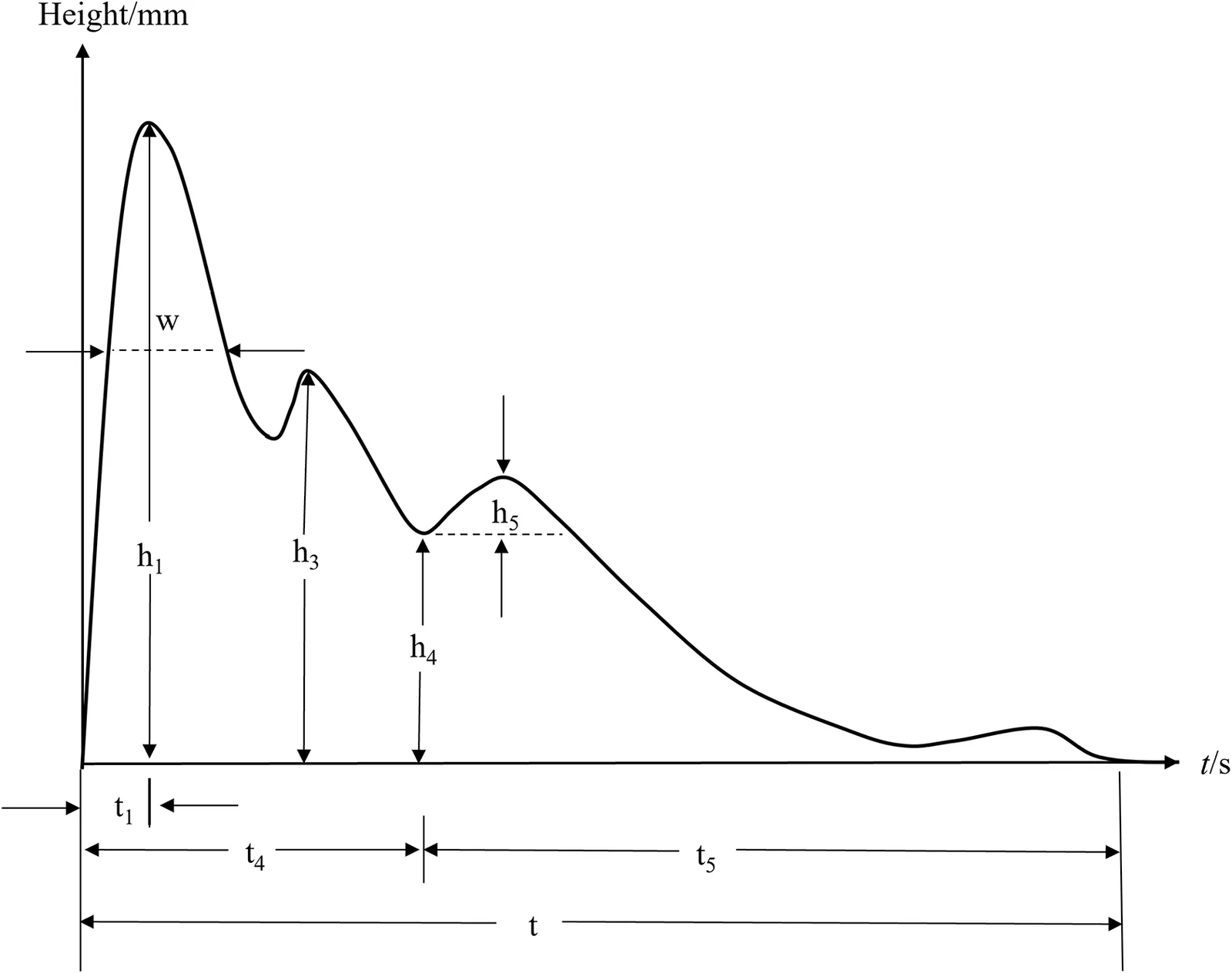
Schematic diagram of the main pulse graph parameters.

**Table 1:** Main parameters and significance of the pulse graph.

Classify	Parameter	Significance
Amplitude	h_1_	Main wave, primarily reflects the compliance of large arteries and the ejection function of the left ventricle
	h_3_	Pre-dicrotic wave, primarily reflects the elastic function of arterial vessels and the state of peripheral resistance
	h_4_	Dicrotic notch, related to peripheral resistance of arterial vessels and the closing function of the aortic valve
	h_5_	Dicrotic wave, related to the compliance of large arteries and the function of the aortic valve
Time value	t	Corresponds to one cardiac cycle of the left ventricle
	t_1_	Corresponds to the rapid ejection period time of the left ventricle
	t_4_	Corresponds to the systolic period time of the left ventricle
	t_5_	Corresponds to the diastolic period time of the left ventricle
Ratio	h_3_/h_1_	Primarily reflects the state of peripheral resistance and the compliance of the vascular wall
	h_4_/h_1_	Primarily reflects the magnitude of peripheral resistance, when the peripheral resistance increases relatively, h_4_/h_1_ increases; otherwise, it becomes smaller
	t_4_/t_5_	Primarily reflects the ratio of the diastolic period to the systolic period of the left ventricle and has a certain relationship with heart rate
	w/t	Proportion of time of sustained high pressure in the aorta within the total pulse cycle

**Table 2:** Main parameters and significance of the cardiovascular ultrasound.

Classify	Parameter (units)	Full title	Significance
Cardiac structure	LVEDD (mm)	Left ventricular end-diastolic dimension	Maximum inner diameter of the left ventricle at end-diastole
	LVESD (mm)	Left ventricular end-systolic dimension	Minimum inner diameter of the left ventricle at end-systole
	IVSd (mm)	Interventricular Septal thickness at diastole	Thickness of the interventricular septum at end-diastole
	LVEDV (mL)	Left ventricular end-diastolic volume	Blood volume in the left ventricle at end-diastole
	LVESV (mL)	Left ventricular end-systolic volume	Residual blood volume in the left ventricle at end-systole
	LVM (g)	Left ventricular mass	Total myocardial mass of the left ventricle
Cardiac function	SV (mL)	Stroke volume	Blood volume ejected by the left ventricle per beat
	EF (%)	Ejection fraction	Percentage of stroke volume relative to end-diastolic volume
Arterial elasticity	cfPWV (m/s)	Carotid-femoral pulse wave velocity	Speed of pulse wave propagation from carotid to femoral artery
	crPWV (m/s)	Common carotid pulse wave velocity	Speed of pulse wave propagation along the common carotid artery.
	PWVρ (m/s)	Pulse wave velocity (density-calibrated)	Pulse wave velocity calibrated by blood density
Vascular structure	IMT (mm)	Carotid intima-media thickness	Distance from the intima-media junction to the adventitia of the far wall of the common carotid artery
Local blood flow velocity	LVAvmax (cm/s)	Left vertebral artery maximum blood flow velocity	Peak systolic blood flow velocity of the left vertebral artery
	LICAvmax (cm/s)	Left internal carotid artery maximum blood flow velocity	Peak systolic blood flow velocity of the left internal carotid artery
	LFAmax (cm/s)	Left femoral artery maximum blood flow velocity	Peak systolic blood flow velocity of the left femoral artery
Vascular resistance	LICA_RI	Left internal carotid artery resistance index	Reflects peripheral resistance of the internal carotid artery bed
	LVA_RI	Left vertebral artery resistance index	Reflects peripheral resistance of the vertebral artery bed
	RFA_RI	Right femoral artery resistance index	Right femoral artery resistance index
	LFA_RI	Left femoral artery resistance index	Reflects peripheral resistance of the femoral artery bed

### 2.4 Methods of statistical analysis

SPSS25.0 software was used to statistically analyze the data, and the normalized data were first tested for normality; the data conforming to the normal distribution were expressed as the mean ± standard deviation (mean ± SD). The concentration and discrete tendency were described, and the data that do not meet the normal distribution are expressed by using the median and interquartile range. For the comparison of the two groups of continuous data, if the normality and variance homogeneity were met, the *t*-test of two independent samples was used for the comparison of the means between the two groups, and the analysis of variance and the LSD test were used for multiple comparisons between multiple independent samples. If normality and variance homogeneity are not met, Wilcoxon rank sum test was used for comparison of means between groups, and Kruskal–Wallis *H*-test was used for comparison between three or more groups. Dunn–Bonferroni test was used for comparison between two groups. Repeated-measures ANOVA was used for multi-time point continuous data, and all tests were two-tailed tests. Statistical significance was set at *P* < 0.05.

## 3 Results

### 3.1 Comparison of pulse graph parameters and cardiovascular ultrasound indexes at baseline, bed rest, and recovery in HDBR experiments

#### 3.1.1 Pulse graph parameters

**Table 3** and **Figure 3** presents the comparison results of pulse graph parameters for all subjects in the HDBR experiment across the three stages: baseline, bedrest, and recovery. The differences in these pulse graph parameters among the three stages were generally statistically significant (*P* < 0.05). Observations indicate that parameters h_1_, h_3_, h_4_, t_5_, t, As, and Ad showed an initial increase followed by a decrease across the three experimental stages. Parameters h_5_, t_4_, and h_5_/h_1_ exhibited an upward trend, while h_3_/h_1_ and w/t showed a downward trend.

**Table 3:** Comparison of the results of three-stage pulse graph parameters (mean ± SD).

Parameters	Basic stage	Bedrest stage	Recovery stage	*F*	*P*
h_1_	17.41 ± 3.18	20.68 ± 4.67^a^	19.40 ± 3.78^a,b^	10.586	<0.001
h_3_	12.02 ± 2.16	13.52 ± 3.59^a^	11.52 ± 3.11^b^	14.488	<0.001
h_4_	6.79 ± 1.62	8.60 ± 2.27^a^	6.51 ± 1.65^b^	44.130	<0.001
h_5_	0.86 ± 2.06	1.00 ± 0.88^a^	1.50 ± 0.83^b^	10.040	<0.001
t_1_	0.11 ± 0.01	0.11 ± 0.01^a^	0.12 ± 0.01^b^	3.399	0.034
t_4_	0.31 ± 0.02	0.34 ± 0.05^a^	0.35 ± 0.03^a,b^	13.653	<0.001
t_5_	0.47 ± 0.09	0.61 ± 0.13^a^	0.55 ± 0.11^a,b^	22.894	<0.001
t	0.79 ± 0.08	0.94 ± 0.12^a^	0.90 ± 0.13^a,b^	25.856	<0.001
As	80.30 ± 17.46	106.37 ± 26.42^a^	95.40 ± 22.33^a,b^	21.430	<0.001
Ad	42.82 ± 14.36	65.80 ± 23.72^a^	46.70 ± 16.81^b^	41.064	<0.001
h_3_/h_1_	0.70 ± 0.12	0.67 ± 0.15	0.60 ± 0.14^a,b^	9.680	<0.001
h_4_/h_1_	0.39 ± 0.07	0.42 ± 0.10	0.34 ± 0.09^a,b^	28.772	<0.001
h_5_/h_1_	0.05 ± 0.11	0.05 ± 0.04	0.08 ± 0.04^a,b^	11.946	<0.001
t_1_/t	0.14 ± 0.02	0.12 ± 0.02^a^	0.13 ± 0.02^a,b^	24.008	<0.001
t_4/_t_5_	0.68 ± 0.20	0.59 ± 0.13^a^	0.67 ± 0.14^b^	17.984	<0.001
w/t	0.18 ± 0.03	0.17 ± 0.05	0.15 ± 0.03^a,b^	13.218	<0.001

^a^ Compared with the basic stage, *P* < 0.05.

^b^ Compared with the bedrest stage, *P* < 0.05.

**Figure 3:**
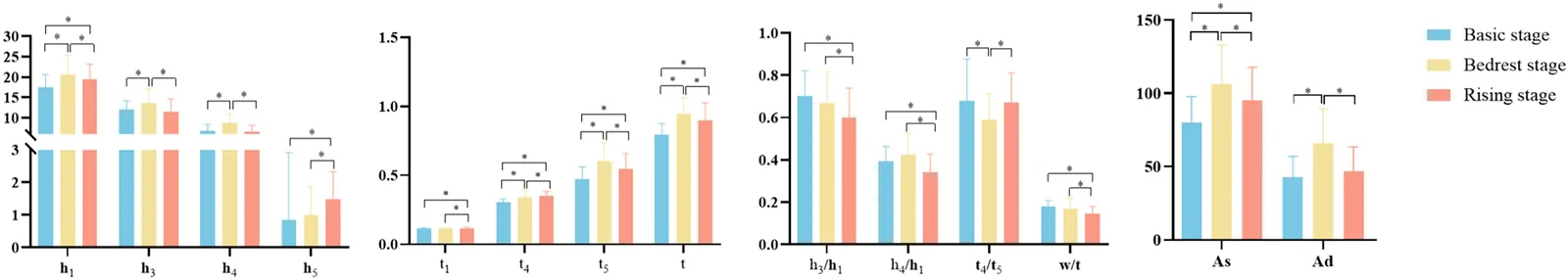
Comparison results of three stages of pulse graph parameters. The asterisk (*) indicates a statistically significant difference between the two groups connected by the bracket (P < 0.05).

#### 3.1.2 Cardiovascular ultrasound indicators

As shown in **Table 4** and **Figure 4**, no statistically significant differences were observed in the cardiac structure and functional parameters among the three stages: baseline, bedrest, and recovery (*P* > 0.05). Vascular elasticity and hemodynamic parameters revealed that PWVρ, crPWV, IMT, LICAvmax, LFAvmax, and LICA_RI showed statistically significant differences across the three experimental stages. Observing the values, PWVρ and crPWV during both bedrest and recovery stages were elevated compared to the baseline period. Carotid IMT showed a continuous increase. LICAvmax and LFAvmax exhibited an initial decrease followed by an upward trend. LICA_RI, LVA_RI, and LFA_RI all demonstrated a continuously decreasing trend.

**Table 4:** Comparison of the results of three-stage cardiovascular ultrasound indicators (mean ± SD).

Parameters	Basic stage	Bedrest stage	Recovery stage	*F*	*P*
EF (%)	65.97 ± 4.42	66.52 ± 4.63	66.69 ± 4.29	0.350	0.705
LVEDD (mm)	45.97 ± 2.96	45.19 ± 3.09	44.92 ± 3.42	1.486	0.228
LVESD (mm)	29.31 ± 2.89	28.63 ± 2.87	28.37 ± 2.64	1.503	0.224
LVEDV (mL)	97.77 ± 14.89	93.74 ± 14.45	92.82 ± 16.50	1.464	0.233
LVESV (mL)	33.57 ± 8.12	31.72 ± 7.85	30.96 ± 7.06	1.578	0.208
LVM (g)	110.52 ± 17.61	105.55 ± 15.45	104.24 ± 16.45^a^	2.095	0.125
SV (mL)	64.21 ± 8.65	62.31 ± 9.91	61.86 ± 11.53	0.705	0.495
cfPWV (m/s)	5.72 ± 0.86	6.01 ± 0.89	5.90 ± 1.10	1.623	0.199
crPWV (m/s)	7.40 ± 1.03	8.14 ± 1.37^a^	8.02 ± 1.26^a^	4.915	0.008
PWVρ (m/s)	5.20 ± 0.59	5.82 ± 0.83^a^	5.87 ± 0.82^a^	10.335	<0.001
IMT (mm)	0.45 ± 0.08	0.50 ± 0.07^a^	0.51 ± 0.07^a^	8.017	<0.001
LICAvmax (cm/s)	53.23 ± 8.59	48.71 ± 7.78^a^	51.58 ± 8.60^b^	7.632	0.001
LVAvmax (cm/s)	43.42 ± 7.38	40.11 ± 7.46^a^	43.82 ± 9.42^b^	8.614	<0.001
LFAvmax (cm/s)	81.20 ± 15.46	67.97 ± 15.08^a^	73.31 ± 15.06^a,b^	13.787	<0.001
LICA_RI	0.57 ± 0.05	0.55 ± 0.05	0.54 ± 0.06^a^	4.021	0.019
LVA_RI	0.65 ± 0.07	0.62 ± 0.06^a^	0.62 ± 0.08^a^	3.313	0.038
LFA_RI	1.36 ± 0.07	1.35 ± 0.09	1.34 ± 0.11	1.349	0.261

^a^ Compared with the basic stage, *P* < 0.05.

^b^ Compared with the bedrest stage, *P* < 0.05.

**Figure 4:**
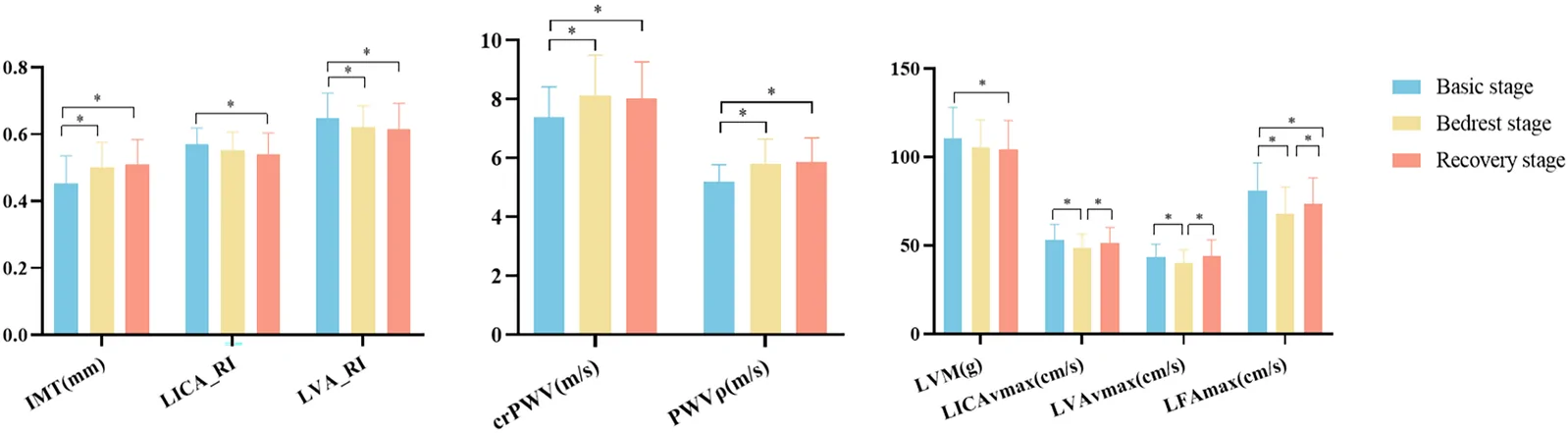
Comparison results of three stages of cardiovascular ultrasound indicators. The asterisk (*) indicates a statistically significant difference between the two groups connected by the bracket (P < 0.05).

### 3.2 Trends of pulse graph parameters and cardiovascular ultrasound indexes in the HDBR control group and intervention group

#### 3.2.1 Pulse graph parameters

**Figure 5** shows that the five groups exhibited generally similar trends in pulse graph parameters. Among amplitude parameters, h_1_ showed a rapid initial increase, followed by a decrease and then a subsequent increase; h_3_ and h_4_ initially increased and then fluctuated downward; h_5_ showed a fluctuating upward trend, especially during the recovery stage when all five groups exhibited a clear increase. Time-domain parameters t_1_, t_4_, t_5_, and t increased initially, then decreased, and increased again. Ratio parameters h_3_/h_1_, h_4_/h_1_, and w/t generally showed a fluctuating downward trend, while h_5_/h_1_ showed a fluctuating upward trend; t_1_/t and t_4_/t_5_ decreased sharply in the early HDBR stage and then gradually increased. Notably, h_3_/h_1_, h_4_/h_1_, w/t, and h_5_/h_1_ maintained similar trends during the recovery stage as observed during HDBR.

**Figure 5:**
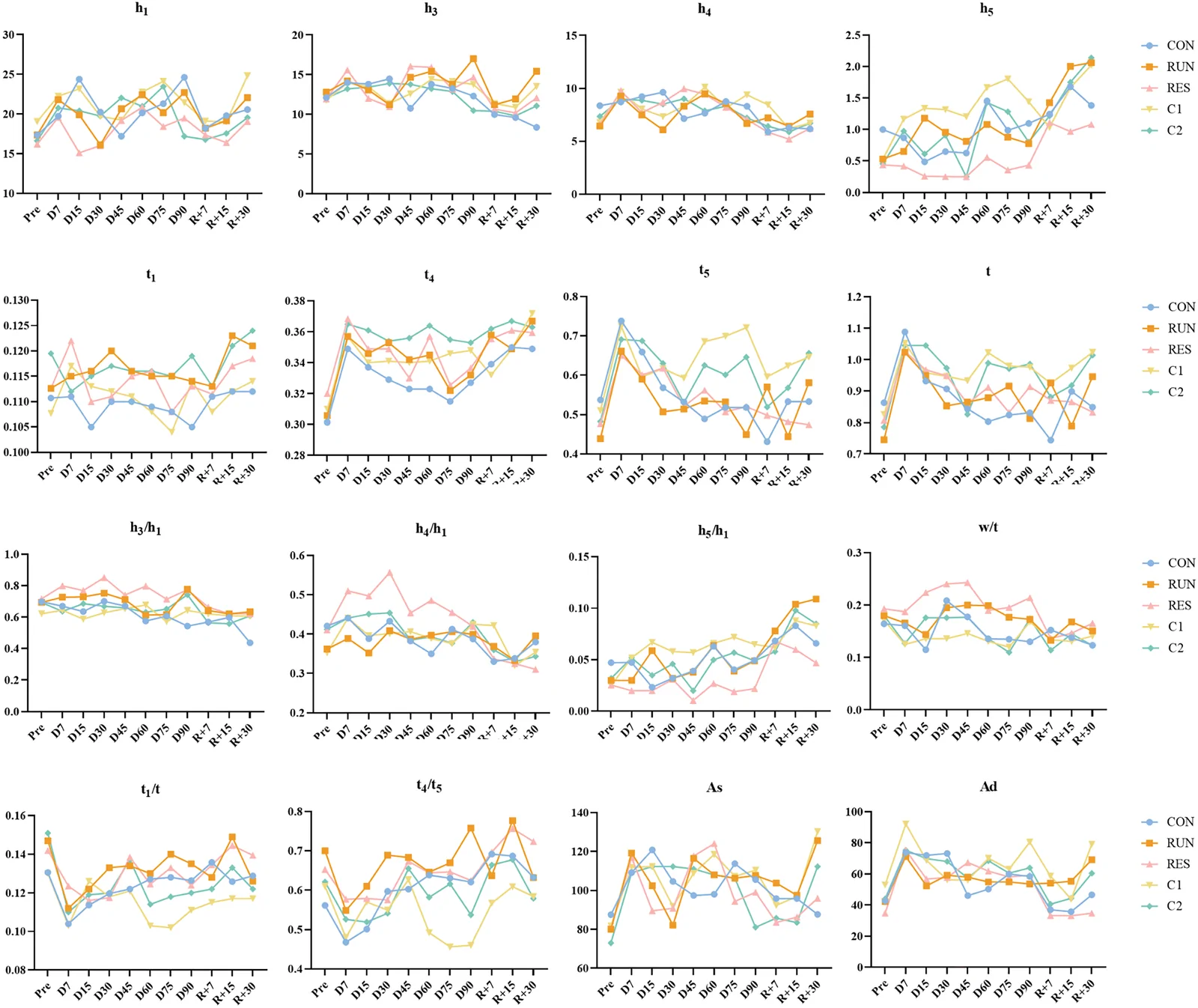
Pulse graph trend of HDBR control group and intervention group at different time points.

Group differences were mainly manifested as follows: the h_1_ peak time occurred earlier in the four intervention groups than in the CON group (e.g., RES group at D7 vs. CON group at D15); the h_5_ and ratio parameter fluctuations in the RES group differed markedly from those in the other four groups; the C2 group exhibited an opposite t_1_ trend at D7 compared to the other groups; and at R + 30, the intervention groups showed the opposite recovery trend to the CON group.

#### 3.2.2 Cardiovascular ultrasound indicators

**Figure 6** shows that the five groups exhibited generally similar trends in cardiovascular ultrasound indicators. The arterial elasticity parameters (cfPWV, crPWV, and PWVρ) and the vascular structural parameter IMT initially increased and then decreased, with D30 and R + 7 as the main turning points. The resistance indices LICA_RI and RICA_RI showed similar patterns, with an overall fluctuating decline followed by a sharp rise after reaching their lowest point at R + 7, whereas the blood flow velocity parameter LFAvmax showed the opposite trend. Regarding group differences in IMT, during early HDBR the CON group showed a continuous increase, whereas the four intervention groups stabilized after an initial rise and then entered a downward trajectory after D30, with the C2 group showing the smallest fluctuations and the RES group the largest. The difference between the CON and C1 groups was most pronounced in the resistance indices, and the CON group exhibited greater fluctuations than the intervention groups across most indicators.

**Figure 6:**
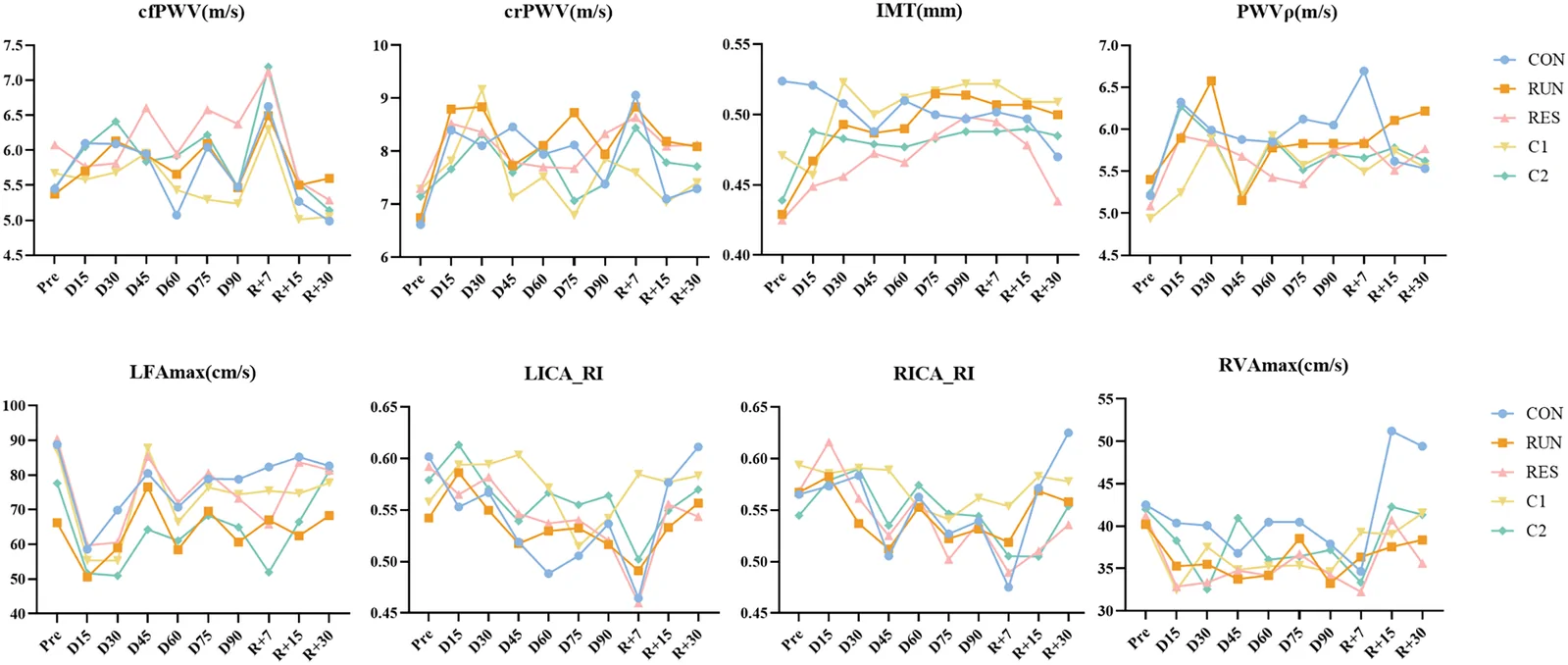
Trend of ultrasound indicators in the HDBR control group and intervention group at different time points.

### 3.3 Study on the overall correlation between pulse graph parameters and cardiovascular ultrasound indicators in HDBR subjects

To comprehensively investigate the relationship between pulse graph parameters and cardiovascular ultrasound indicators, this study employed both canonical correlation analysis and bivariate correlation analysis to evaluate their correlations. Canonical correlation analysis was used to explore whether the “pulse graph parameter set” and the “cardiovascular ultrasound indicator set” are intrinsically associated as two wholes, while bivariate correlation analysis was applied to identify one-to-one linear associations between specific indicators.

The canonical correlation analysis revealed a moderate to high correlation between pulse graph parameters and cardiovascular ultrasound indicators during long-term HDBR (C = 0.741, 0.674; *P* < 0.05). Among the two sets of variables, cardiovascular ultrasound indicators contributed more significantly, particularly the carotid and femoral artery resistance indices and cfPWV. Pulse graph parameters with higher contribution weights included h_3_, h_4_, t_5_, As, h_3_/h_1_, h_5_/h_1_, and w/t. Bivariate correlation analysis between pulse graph parameters and cardiovascular ultrasound indicators at different time points during HDBR showed varying degrees of correlation between the two types of measurements. In the baseline state, pulse parameters h_3_/h_1_, h_4_/h_1_, t_1_/t, w/t, t_4_, t_5_, and t correlated with aortic resistance, cardiac ejection fraction, cardiac output, and ventricular dimensions. For the bivariate correlation analysis, we selected correlations with coefficients greater than 0.3 that were maintained across four or more time points. The most prominent pulse graph parameters were t, t_5_, h_3_, h_5_, w/t, and t_1_/t, while the most significant cardiovascular ultrasound parameters were cfPWV, PWVρ, LICA_RI, and LFA_RI. These findings are generally consistent with the canonical correlation results described above (**Figures 7**, **8)**.

**Figure 7:**
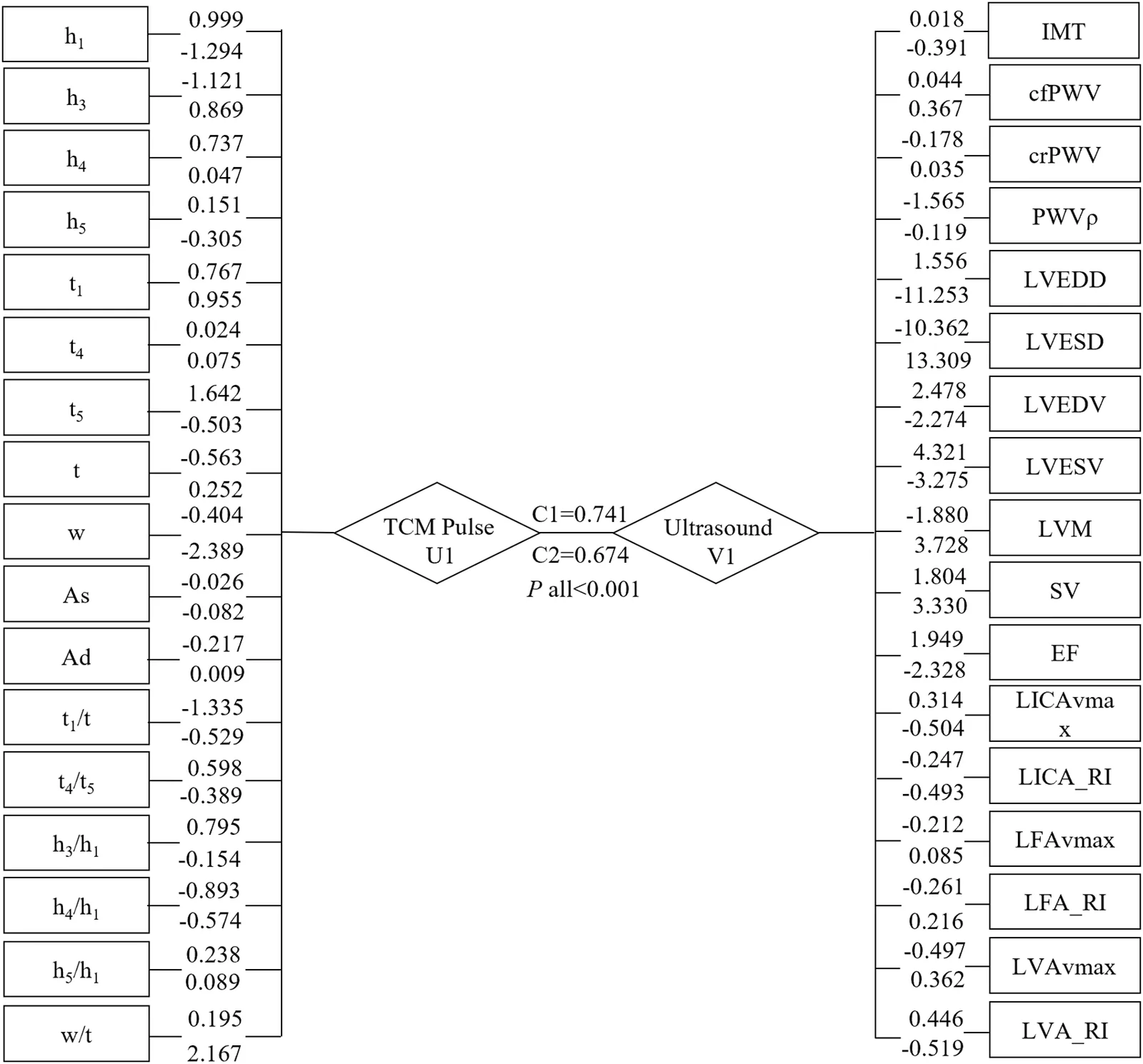
Typical correlation results between pulse graph parameters and cardiovascular ultrasound indicators. The 10 time points of the HDBR experiment (basic stage; days 15, 30, 45, 60, 75, and 90 of bedrest stage; days 7, 15, and 30 of recovery stage) were used as typical correlations. A total of four sets were statistically significant, and the correlation coefficients of the two highest ensemble variables were selected in this image, and the correlation coefficients and statistical results of the rest were 0.619 and 0.517, *P* < 0.05, respectively.

**Figure 8:**
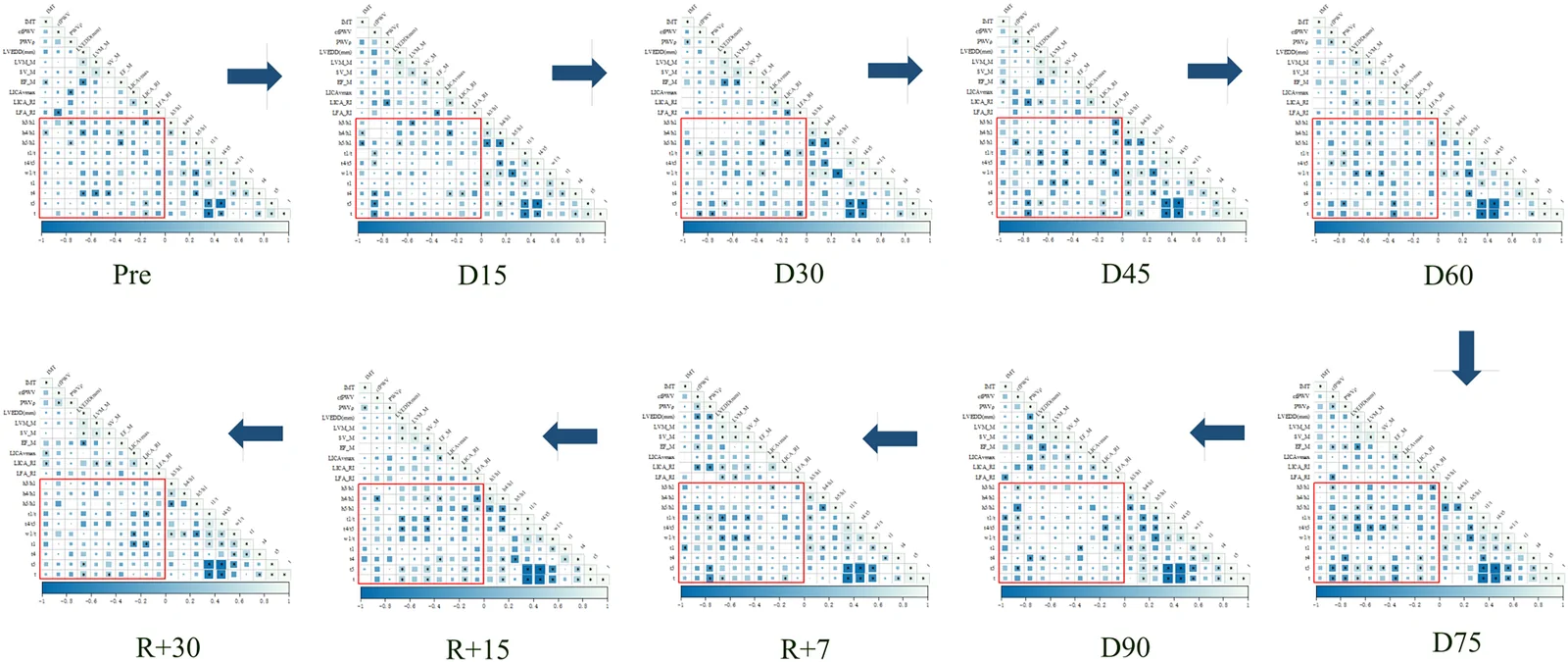
Bivariate correlation results between pulse graph parameters and cardiovascular ultrasound indicators at different time points.

### 3.4 Repeated-measures results of pulse graph parameters and cardiovascular ultrasound indicators at multiple time points in the HDBR experiment

#### 3.4.1 Pulse graph parameters

**Table 5** and **Figure 9** indicates that prolonged -6° HDBR significantly affected the amplitude, time, and ratio parameters of the pulse graph in the subjects, with statistically significant differences across multiple time points in repeated-measures analysis (*P* < 0.05). As shown in **Figure 9a**, the changes in h_1_, h_3_, and h_4_ were generally consistent across multiple time points, showing an increase from the baseline period to the early bedrest period (day 7), a decrease followed by an increase during the mid-bedrest period (days 15 to 60), and a decrease followed by a slight increase or maintenance during the late bedrest period (days 75 and 90), whereas h_5_ showed an overall slightly fluctuating increase. However, with the continuation of the head-down position, h_1_, h_3_, and h_5_ show a fluctuating rise, peaking on day 60 before declining. Upon recovery to the normal state (days 7 to 30 after rising), h_1_, h_3_, and h_4_ initially decreased then increased, while h_5_ increased; among these, h_1_ and h_5_ remained significantly different from the baseline period on day 30.

**Table 5:** Repeated measures ANOVA results for pulse graph parameters at multiple time nodes.

Index	Pre	D7	D15	D30	D45	D60	D75	D90	R+7	R+15	R+30	F	P
h_1_	17.41±3.18	21.04±3.94	20.05±4.58	19.44±6.34	21.20±4.74	21.94±4.23	20.73±3.86	20.33±4.49	18.21±3.32	18.56±2.32	21.42±4.57	5.764	< 0.001
h_3_	12.02±2.16	13.87±3.10	12.95±2.24	12.66±4.65	14.19±3.99	14.36±3.44	13.17±2.52	13.42±4.41	11.09±2.60	10.72±2.27	12.76±3.91	6.191	< 0.001
h_4_	6.79±1.62	9.26±1.96	8.27±1.88	8.40±3.29	8.75±2.18	8.97±2.32	8.26±1.63	8.31±2.22	6.72±1.66	5.90±1.61	6.93±1.54	14.175	< 0.001
h_5_	0.53±0.64	0.86±0.69	1.03±0.83	0.90±0.95	0.79±1.03	1.33±0.92	1.14±0.91	0.97±0.70	1.25±0.67	1.60±0.86	1.63±0.91	10.613	< 0.001
t_1_	0.11±0.01	0.12±0.01	0.11±0.01	0.12±0.01	0.11±0.01	0.11±0.01	0.11±0.01	0.11±0.01	0.11±0.01	0.12±0.01	0.12±0.01	3.595	0.001
t_4_	0.31±0.02	0.36±0.03	0.35±0.03	0.34±0.03	0.34±0.02	0.35±0.02	0.34±0.02	0.32±0.13	0.35±0.04	0.35±0.02	0.36±0.03	5.106	0.015
t_5_	0.47±0.09	0.69±0.10	0.63±0.12	0.60±0.12	0.55±0.11	0.60±0.13	0.58±0.12	0.59±0.17	0.54±0.12	0.53±0.12	0.58±0.10	15.227	< 0.001
As	80.30±17.46	112.91±30.56	105.59±21.45	98.80±33.47	108.32±24.72	114.72±24.76	103.13±21.09	101.14±24.60	88.69±19.79	89.89±15.49	107.63±25.70	9.417	< 0.001
Ad	42.82±14.36	82.00±28.84	65.18±20.10	60.75±24.02	61.82±21.81	63.85±21.37	61.76±18.76	65.27±24.54	43.02±12.96	41.46±16.10	55.62±17.69	18.239	< 0.001
t	0.79±0.08	1.05±0.11	0.99±0.10	0.94±0.10	0.88±0.10	0.94±0.12	0.90±0.12	0.91±0.12	0.89±0.14	0.88±0.14	0.93±0.10	21.477	< 0.001
h_3_/h_1_	0.70±0.12	0.67±0.14	0.66±0.12	0.67±0.18	0.68±0.15	0.66±0.15	0.64±0.11	0.67±0.22	0.62±0.14	0.58±0.12	0.60±0.16	2.91	0.005
h_4_/h_1_	0.39±0.07	0.45±0.09	0.43±0.12	0.45±0.14	0.42±0.09	0.41±0.09	0.40±0.08	0.42±0.10	0.37±0.08	0.32±0.09	0.33±0.08	9.318	< 0.001
h_5_/h_1_	0.03±0.04	0.04±0.03	0.05±0.04	0.05±0.05	0.04±0.05	0.06±0.04	0.05±0.04	0.05±0.03	0.07±0.04	0.09±0.05	0.08±0.04	10.881	< 0.001
t_1_/t	0.14±0.02	0.11±0.01	0.12±0.01	0.12±0.02	0.13±0.02	0.12±0.02	0.13±0.02	0.13±0.02	0.13±0.02	0.14±0.02	0.13±0.02	14.679	< 0.001
t_4_/t_5_	0.68±0.20	0.52±0.08	0.57±0.12	0.59±0.11	0.64±0.10	0.60±0.13	0.60±0.10	0.59±0.19	0.67±0.14	0.70±0.15	0.64±0.12	7.175	< 0.001
w/t	0.18±0.03	0.16±0.05	0.16±0.04	0.18±0.05	0.19±0.05	0.16±0.05	0.16±0.05	0.18±0.05	0.14±0.04	0.15±0.03	0.15±0.04	6.594	< 0.001

**Figure 9:**
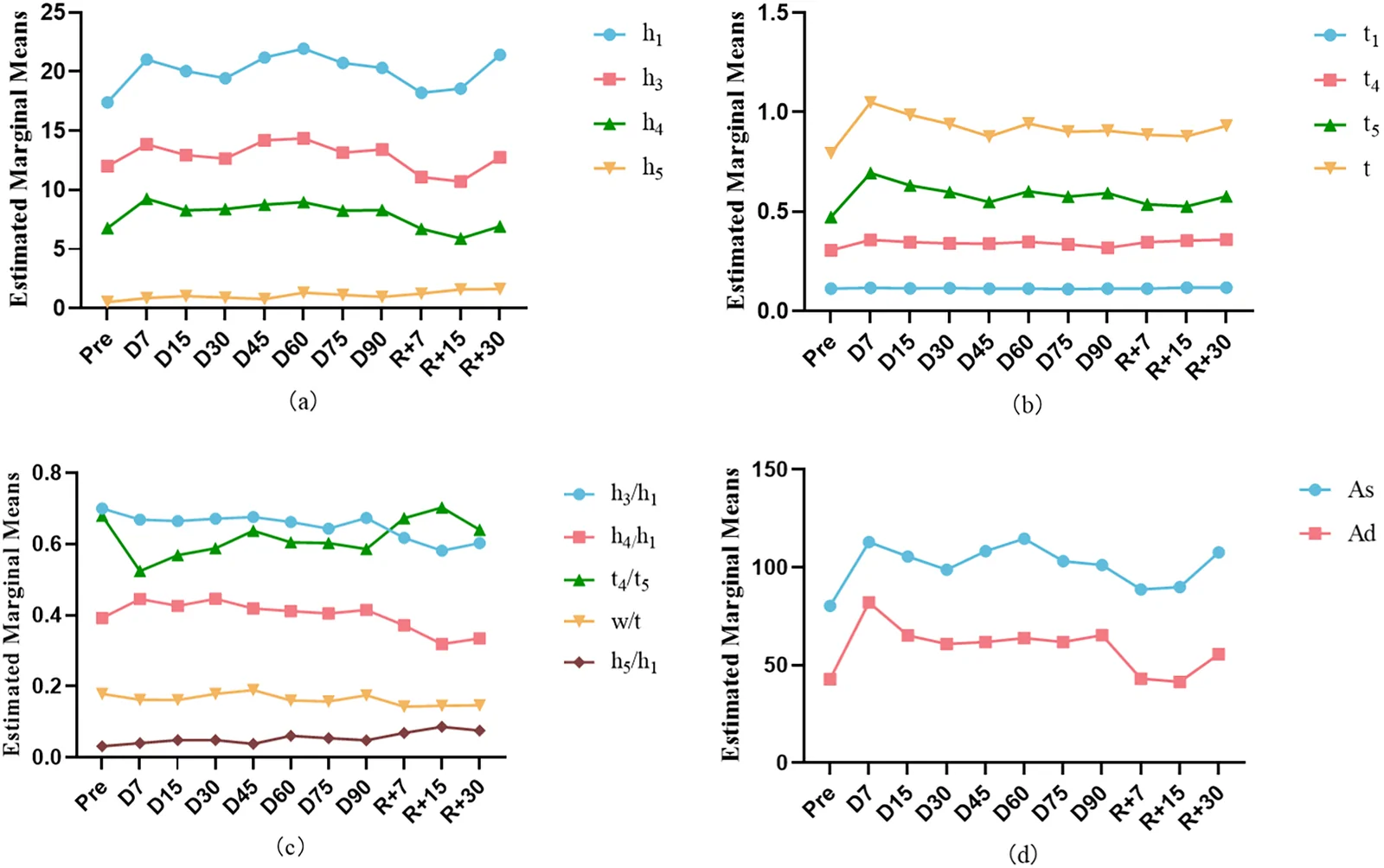
**(a)** Estimated marginal means of amplitude parameters (h_1_, h_3_, h_4_, h_s_). **(b)** Estimated marginal means of time-domain parameters (t_1_, t_4_, t_s_, t). **(c)** Estimated marginal means of ratio parameters (h₃/h₁, h₄/h₁, t₄/t₅, w/t, h₅/h₁). **(d)** Estimated marginal means of As and Ad. Each line is color-coded and marked in the legend. The x-axes represent time points from Pre to R+30 days.

Among the ratio parameters, h_3_/h_1_ and w/t showed an overall fluctuating downward trend, h_5_/h_1_ showed an overall fluctuating upward trend, h_4_/h_1_ increased in the early bedrest period and then decreased, and t_1_/t and t_4_/t_5_ decreased sharply in the early bedrest period and then gradually recovered. After entering the recovery period, h_3_/h_1_, h_4_/h_1_, and w/t all decreased compared with the baseline period, whereas h_5_/h_1_ increased compared with both the baseline and bed rest periods.

#### 3.4.2 Cardiovascular ultrasound indicators

As shown in the **Table 6** and **Figure 10**, cfPWV, crPWV, and PWVρ all increase during the early stage of the HDBR experiment, followed by a tortuous decline. cfPWV and PWVρ exhibit nearly identical trends, and all three parameters show an increase on day 7 of recovery. The cardiac ultrasound indicators remain relatively stable, with a notable decrease observed after the day 90 bedrest period upon return to normal gravity (e.g., LVEDD and LVESD).

**Table 6:** Repeated measures ANOVA results of some parameters of cardiac ultrasound at multiple time nodes.

Index	Pre	D15	D30	D45	D60	D75	D90	R+7	R+15	R+30	F	P
IMT (mm)	0.45±0.08	0.48±0.07	0.50±0.08	0.50±0.08	0.50±0.07	0.52±0.07	0.52±0.07	0.52±0.07	0.51±0.07	0.49±0.07	15.034	< 0.001
cfPWV (m/s)	5.72±0.86	6.03±0.86	6.09±0.79	6.37±1.21	5.79±0.74	6.06±0.75	5.70±0.82	6.82±1.13	5.52±0.80	5.36±0.69	17.641	< 0.001
crPWV (m/s)	7.40±1.03	8.39±1.31	8.54±1.35	7.95±1.47	8.02±1.35	7.79±1.22	8.12±1.44	8.60±1.37	7.64±1.15	7.82±1.04	6.042	< 0.001
PWVθ (m/s)	5.20±0.59	5.96±0.81	6.08±0.95	5.61±0.65	5.79±0.98	5.72±0.85	5.75±0.66	6.05±0.89	5.78±0.73	5.79±0.83	6.612	< 0.001
LVEDD (mm)	45.97±2.96	45.94±2.64	45.44±2.93	45.11±2.87	45.17±3.75	44.86±3.05	44.64±3.24	42.58±3.37	45.56±2.76	46.61±2.79	20.267	< 0.001
LVESD (mm)	29.31±2.89	29.06±2.55	28.89±2.94	28.58±3.00	28.50±2.96	28.56±2.89	28.17±2.98	27.42±2.80	28.31±2.21	29.39±2.56	5.393	< 0.001
IVSd (mm)	7.64±0.68	7.50±0.56	7.61±0.55	7.64±0.68	7.67±0.72	7.67±0.59	7.65±0.58	7.60±0.55	7.60±0.55	7.69±0.64	0.873	0.549
LVEDV (mL)	97.77±14.89	97.52±13.33	95.16±14.76	93.52±14.08	92.46±14.31	92.38±14.69	91.40±15.55	81.92±15.78	95.64±13.70	100.90±14.07	22.597	< 0.001
LVESV (mL)	33.57±8.12	32.77±7.15	32.44±8.28	31.64±8.24	31.41±8.10	31.53±7.79	30.53±7.82	28.54±7.42	30.65±5.82	33.68±7.06	5.083	< 0.001
LVM (g)	110.52±17.61	106.96±15.18	106.76±15.56	106.01±16.12	105.24±15.36	104.82±16.14	103.48±15.13	94.94±15.05	106.27±14.36	111.50±15.71	13.342	< 0.001
SV (mL)	64.21±8.65	64.75±9.75	62.72±8.34	61.88±8.26	62.77±14.08	60.86±8.78	60.87±9.17	53.38±9.95	64.99±10.67	67.22±8.96	14.927	< 0.001
EF (%)	65.97±4.42	66.47±4.88	66.24±4.53	66.50±5.06	66.77±4.76	66.17±4.50	66.94±4.32	65.37±4.34	67.92±4.32	66.79±3.91	1.601	0.147
LVAVmax (cm/s)	43.42±7.38	38.73±7.65	40.46±7.81	39.82±7.70	41.10±6.88	39.71±7.03	40.86±7.93	40.85±8.39	45.27±8.47	45.35±10.74	6.426	< 0.001
LICAVmax (cm/s)	53.23±8.59	46.38±6.39	47.88±8.71	50.55±7.46	49.45±7.90	48.58±6.89	49.40±8.89	46.45±7.43	55.79±7.38	52.50±8.37	8.339	< 0.001
LFAMax (cm/s)	81.20±15.46	56.43±9.84	59.95±10.68	80.27±15.81	65.32±13.08	76.69±13.00	69.18±12.26	69.00±15.59	74.48±13.61	76.46±15.31	24.683	< 0.001
LICA_RI	0.57±0.05	0.58±0.05	0.58±0.05	0.54±0.06	0.55±0.05	0.53±0.04	0.54±0.04	0.50±0.07	0.55±0.05	0.57±0.05	10.353	< 0.001
LVA_RI	0.65±0.07	0.64±0.06	0.62±0.08	0.62±0.06	0.62±0.06	0.61±0.06	0.62±0.07	0.57±0.08	0.62±0.06	0.66±0.07	9.585	< 0.001
RFA_RI	1.37±0.07	1.42±0.08	1.38±0.08	1.34±0.07	1.38±0.09	1.34±0.07	1.39±0.08	1.34±0.06	1.33±0.12	1.39±0.07	6.469	< 0.001
LFA_RI	1.36±0.07	1.35±0.09	1.36±0.07	1.34±0.08	1.38±0.11	1.33±0.07	1.35±0.09	1.33±0.06	1.31±0.13	1.37±0.12	2.439	0.030

**Figure 10:**
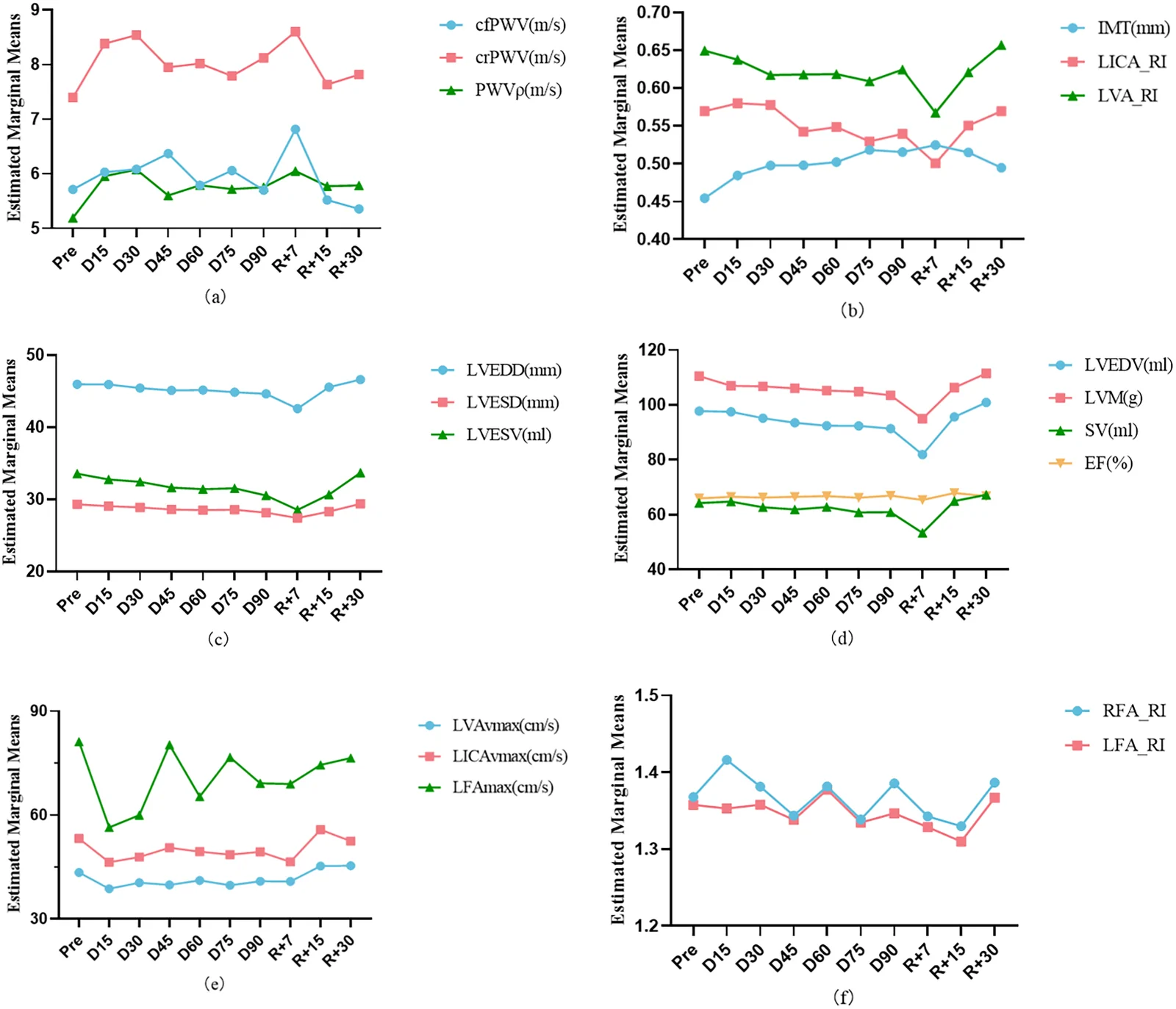
**(a)** Estimated marginal means of pulse wave velocity parameters (cfPWV, crPWV, PWVp). **(b)** Estimated marginal means of vascular structure and resistance indices (IMT, LICA_RI, LVA_RI,). **(c)** Estimated marginal means of cardiac structure (LVEDD, LVESD, LVESV). **(d)** Estimated marginal means of cardiac structure and function (LVEDV, LVM, SV, EF). **(e)** Estimated marginal means of local blood flow velocity (LFAvmax, LICAvmax, LVAvmax). **(f)** Estimated marginal means of resistance indices(RFA_RI, LFA_RI) . Each line is color-coded and marked in the legend. The x-axes represent time points from Pre to R+30 days.

## 4 Discussion and conclusion

Pulse diagnosis is a distinct diagnostic method in traditional Chinese medicine (TCM). With the advancement of modern technology, research on TCM pulse diagnosis has entered an era of modernization and informatization. The development of digital pulse diagnosis technology provides a novel non-invasive approach for examining cardiovascular diseases (Cui and Xu, 2023; Wang et al., 2023; Huang et al., 2024). Huang JL et al (Huang et al., 2024). collected pulse graph parameters from 550 individuals with isolated systolic hypertension (ISH) and found correlations between these parameters and age and blood pressure gradients, offering reference value for clinical diagnosis and risk assessment of ISH. Wu WJ et al (Wu et al., 2023). used an ECG-pulse pressure multi-source sensor pulse diagnostic instrument to synchronously collect pulse and ECG signals from patients with coronary heart disease and found that the time-domain characteristics of patients’ pulses can reflect the changes in ECG signal characteristics to a certain extent. The research of TCM pulse diagnosis has achieved certain results not only in cardiovascular diseases but also in other clinical diseases, suggesting that TCM pulse diagnosis may provide auxiliary reference for the diagnosis of diseases.

In this study, the pulse parameters of HDBR subjects showed significant differences across the three stages: baseline, bedrest, and recovery. During bedrest stage, postural changes induced body fluid redistribution. According to previous studies, this change can lead to a short-term increase in venous return (Li et al., 2016). To adapt to this redistribution, blood vessels dilated, peripheral resistance decreased, and stroke volume increased (Li et al., 2016). Upon entering the recovery stage, blood flow redistributed back, and both pulse parameters and cardiovascular ultrasound indicators changed correspondingly.

Previous studies have shown that pulse wave velocity (PWV) increases after HDBR, suggesting increased vascular stiffness (Boutouyrie et al., 2022), while other studies have not observed significant changes in vascular stiffness parameters. In the present study, cfPWV, which reflects aortic stiffness, remained relatively stable, whereas PWVp and crPWV showed higher mean values during the bedrest and recovery periods compared with the baseline period. These results suggest that different arteries may respond differently to HDBR, possibly related to exercise intervention, the relatively long experimental duration, and the absence of pharmacological intervention. LICAvmax and LFAvmax showed trends consistent with the pulse parameter h_3_/h_1_, suggesting that postural changes have a notable impact on local hemodynamics. LICA_RI, LVA_RI, and LFA_RI all showed a sustained decreasing trend, with the most pronounced changes observed in LICA_RI, suggesting more active regulatory responses in carotid vascular resistance. This finding is consistent with the decreasing trend of w/t, suggesting a possible overall reduction in peripheral vascular resistance, as well as adaptive adjustments of the cardiovascular system.

A comparison of different exercise intervention modalities revealed that the h_1_ peak appeared earlier in the exercise intervention groups than in the control group, suggesting that exercise intervention may help maintain venous return and cardiac preload and delay the decline in cardiac function induced by weightlessness. The increases in h_3_ and h_4_ in the RES group may be related to acute vasoconstrictive responses induced by resistance training, while the fluctuating changes in h_5_ suggest that different exercise modalities exert differential effects on aortic compliance. Under HDBR conditions, left ventricular systolic function recovery may precede diastolic function recovery, with HDBR combined with high-load resistance training showing a more pronounced effect on cardiac ejection function in the early experimental stage. Among the ratio parameters, h_3_/h_1_, h_4_/h_1_, w/t, and h_5_/h_1_ maintained trends similar to those observed during HDBR in the recovery period. This phenomenon is related to cardiovascular readaptation after the transition from head-down position to normal posture, and its potential causes include hypovolemia, increased vascular compliance, and cardiac atrophy (Aubert et al., 2005; Harris et al., 2022). The short-term increases in IMT and local PWV may be related to changes in vascular wall stress caused by headward fluid shift, and whether they represent permanent stiffening requires further verification through long-term follow-up. Exercise intervention may slow this change by maintaining endothelial function. The resistance indices LICA_RI and RICA_RI showed trends opposite to that of LFA_max, suggesting that local blood flow velocity is considerably influenced by posture, which is also consistent with the view that exercise intervention may have a protective effect against weightlessness-induced cardiovascular deconditioning. Meanwhile, the observed intergroup trends suggest that aerobic exercise combined with appropriate resistance training may help improve adaptability during the experiment and may be more beneficial for the recovery of cardiovascular function after the experiment. It should be noted that the intergroup comparisons in this study are based on trend observations at each time point, mainly reflecting the overall direction of changes in pulse diagram parameters and cardiovascular ultrasound indicators under different exercise interventions, which may provide new ideas for subsequent research.

Furthermore, this study employed canonical correlation analysis and bivariate correlation analysis to verify the correlation between pulse graph parameters and cardiovascular ultrasound indicators at both the “global” and “specific” levels. The two types of indicators showed moderate correlations in canonical correlation analysis, and bivariate correlation analysis also revealed varying degrees of correlation at each time point. This suggests that the effects of HDBR on human cardiovascular function, especially changes in vascular elasticity and blood flow, can be correspondingly reflected in pulse diagram parameters.

Repeated-measures results showed that the changing trends of h_1_, h_3_, h_4_, and h_5_ suggest that HDBR-simulated weightlessness can induce stress-related changes in the cardiovascular system, while the changing trends of the ratio parameters suggest that the cardiovascular state enters an adaptive process after entering the long-term bedrest phase, but a certain period of time is still needed during the recovery period to gradually recover to the baseline state before bedrest. Moreover, h_5_/h_1_ was elevated compared with the baseline and bedrest periods. According to modern traditional Chinese medicine research (Fei, 2003), h_5_ and h_5_/h_1_ are related to large artery compliance and aortic valve function. Although this study did not directly assess aortic valve structure and function, it may preliminarily reflect that the effects of long-term HDBR on different parts of the cardiovascular system differ, and its readaptation may require phased regulation. Cardiac ultrasound structural indicators (LVEDD and LVESD) showed a marked decline after the 90-day bedrest concluded, and normal gravity was resumed, suggesting that cardiac structure and function may be in an unstable state after prolonged HDBR. This change is consistent with the mechanism of reduced venous return after postural changes; however, since this study did not directly measure venous return or central venous pressure, this interpretation remains inferential, suggesting that protective measures are necessary. Through dynamic monitoring of pulse diagram parameters, regular changes in pulse wave characteristics can be observed in volunteers during the HDBR experiment. These changes are consistent with the trends of some cardiovascular ultrasound indicators and align with the TCM diagnostic concept of “pulse manifestations corresponding to time.” Cardiovascular ultrasound can quantitatively assess indicators such as cardiac structure, function, and arterial elasticity, providing a hemodynamic reference for changes in pulse diagram parameters. Combining the two approaches helps to understand the characteristics of cardiovascular changes during HDBR from different perspectives and provides reference ideas for the comprehensive assessment of human functional status under simulated weightlessness in future studies.

In summary, cardiovascular changes induced by HDBR can be correspondingly reflected in pulse parameters. During bedrest, pulse parameters such as h_1_, h_3_, h_4_, and h_5_, as well as PWVρ and crPWV, showed stress-induced elevations, with most parameters reaching a turning point around day 60; during the recovery period, the ratio parameters and cardiovascular indicators suggest the presence of a cardiovascular readaptation process. Combined aerobic and resistance exercise may have potential advantages in maintaining venous return and delaying weightlessness-induced cardiac function decline, but further verification is needed. It should be noted, however, that HDBR is a ground-based simulation and cannot fully replicate the combined effects of multiple stressors present in the actual space environment, such as radiation, isolation, and circadian rhythm disruption. Furthermore, all participants were healthy young men, and the generalizability of the findings to women and other age groups requires further validation. Future studies should examine the assessment efficacy of pulse diagnosis technology in more complex environmental simulations and extend the research to broader populations.

Overall, integrating ultrasound technology with TCM pulse diagnosis helps to assess the functional status of the cardiovascular system during HDBR from different perspectives. This provides new ideas for the comprehensive assessment of human functional status under simulated weightlessness in future studies and also reflects the potential application value of integrated traditional Chinese and Western medicine approaches in the field of space medicine.

## Data Availability

The original contributions presented in the study are included in the article/supplementary material. Further inquiries can be directed to the corresponding authors.
